# Radiofrequency ablation of supraventricular tachyarrhythmias in newborns and infants: why, when, and how?

**DOI:** 10.1007/s12471-025-01985-w

**Published:** 2025-09-17

**Authors:** Andreia Palma, Robin A. Bertels, Marta de Riva, Katja Zeppenfeld, Nico A. Blom

**Affiliations:** 1 Willem-Alexander Children’s Hospital, Leiden University Medical Centre, Leiden, The Netherlands; 2 Paediatric Cardiology Department, Coimbra’s Hospital and University Centre, Coimbra, Portugal; 3 Department of Cardiology, Leiden University Medical Centre, Leiden, The Netherlands; 4 Emma Children’s Hospital, Amsterdam UMC, Amsterdam, The Netherlands

**Keywords:** Radiofrequency catheter ablation, Supraventricular tachyarrhythmias, Accessory pathways, Infants, Neonates

## Abstract

Supraventricular tachyarrhythmias (SVTs) in newborns and infants (< 1 year) can usually be managed with antiarrhythmic drug therapy and often resolve spontaneously within the first year of life. However, some SVTs may be drug-refractory, leading to hemodynamic instability or tachycardia-induced cardiomyopathy, necessitating catheter ablation. Refinements in ablation techniques like electro-anatomical mapping and increased expertise have made catheter ablation an effective option for young children (< 5 years). Nonetheless, due to a lack of large-scale studies involving newborns and infants (< 1 year), concerns persist regarding the risk of major complications associated with ablation energy in small hearts. Potential risks include coronary artery lesions, valve damage, vessel injury, complications related to the transseptal puncture, and ablation lesion growth. In this review, we discuss the current literature on the indications, techniques, and outcomes of radiofrequency catheter ablation in newborns and infants, illustrated by recent cases.

**Supplementary Information:**

The online version of this article (10.1007/s12471-025-01985-w) contains supplementary material, which is available to authorized users.

## Introduction

Supraventricular tachyarrhythmias (SVT) are relatively common in newborns and infants (< 1 year), occurring in approximately 22 per 100,000 live births, with atrioventricular re-entrant tachycardias (AVRT) being the most prevalent subtype [1]. Diagnosing SVTs in infants can be challenging, as they often present with non-specific symptoms such as poor feeding, irritability, lethargy, shortness of breath, and vomiting, which are common among various conditions [2, 3]. If a SVT goes unrecognized for hours to days, the infant may develop progressive heart failure symptoms or cardiovascular collapse, at which point the SVT becomes life-threatening. Over 50% of affected infants present with heart failure upon diagnosis [4].

Radiofrequency catheter ablation (RFCA) is rarely performed in infants, comprising only about 1% of paediatric RFCA procedures, as medical therapy is usually effective and approximately one-third of AVRTs resolve spontaneously within the first year of life [2, 5, 6]. Consequently, current guidelines indicate that RFCA is only appropriate for life-threatening and drug-resistant tachyarrhythmias in young children (< 5 years) after the failure of various combinations of antiarrhythmic drugs (AAD), including Class I and Class III AAD therapy [7, 8]. Data on the efficacy and safety of RFCA in newborns and infants (< 1 year), is limited, raising concerns about potential complications such as atrioventricular block, coronary artery lesions, valve damage, vessel damage, and transseptal puncture related complications [8–12].

In this report, we present six consecutive infants, aged 12 months or younger, who underwent RFCA for drug-resistant SVT or tachycardia-induced cardiomyopathy (TIC), and we review the literature on the indications, technical challenges, and outcomes associated with this procedure.

## RFCA in newborns and infants: case series

Between January 2023 and June 2024, six consecutive infants (< 1 year), weighing 3.1–7.8 kg, underwent RFCA procedures for SVT at the Leiden University Medical Centre, Centre for Congenital Heart Disease (CHD) Amsterdam-Leiden. Patient data are summarized in Tab. 1.

**Table 1:** Pre-ablation cardiac status and clinical findings of patients

Patient number	1	2	3	4	5	6
Sex	M	M	F	M	F	M
Age at diagnosis	Fetal (32 w)	Fetal (34 w)	1 w	5 w	6 w	4 m
Age at RFCA (months)	1.5 (re-do 2)	1 (re-do 2)	3.5	1.8	1.5	12
Weight at RFCA (Kg)	4.5 (re-do 5)	3.1 (re-do 4.4)	7.5	4	4	7.8
CHD	No	No	Ebstein	No	No	MV stenosis & multiple VSD
LVEF	Normal	40%	Normal	< 20%	< 20%	Normal
AAD	Amiodarone Flecainide Digoxin Sotalol	Amiodarone Flecainide Propranolol	Amiodarone Flecainide Sotalol	Amiodarone Flecainide Sotalol	Digoxin Ivabradine Esmol	Flecainide Ivabradine Esmol
Cycle length at presentation	260	290	250	260	230	240
Cycle length under AAD	400	390	380	320	280	400
RFCA indication	Refractory SVT	Refractory SVT	Refractory SVT	TIC	TIC	Refractory SVT
SVT mechanism/substrate	Right lateral AP	Left posterior AP	Right posterior AP	Left lateral AP	Focal AT LAA	CTI dependent AFL & Micro re-entrant AT

*AADs* antiarrhythmic drugs, *ASD* atrial septal defect, *F* female, *Kg* Kilograms, *LVEF* left ventricle ejection fraction, *m* months, *M* male, *MPA* main pulmonary artery, *MV* mitral valve, *RFCA* radiofrequency catheter ablation, *TTE* transthoracic echocardiogram, *VSD* ventricular septal defect, *w* weeks

### Clinical presentation

*Patient 1* (male) was diagnosed with AVRT prenatally at 32 weeks, which converted to sinus rhythm (SR) with maternal digoxin therapy. Postnatally, the ECG showed ventricular preexcitation suggestive of a right-sided accessory pathway (AP), which later resolved. However, AVRT recurred ten days after birth, becoming incessant despite various AAD combinations including digoxin, sotalol, flecainide, and amiodarone, resulting in only short periods of SR following adenosine administration.

*Patient 2* (male) was also diagnosed prenatally with AVRT and was born at 35 weeks in tachycardia despite maternal digoxin therapy. After birth, he experienced nearly incessant tachycardia that persisted despite triple AAD therapy.

*Patient 3* (female) was prenatally diagnosed with severe Ebstein’s anomaly. Postnatally, the ECG showed ventricular preexcitation suggestive of a right posterior AP. She presented one week after birth with fast AVRT, which was hemodynamically poorly tolerated. The AVRT responded to adenosine, but it frequently recurred despite various AAD combinations.

*Patient 4* (male) presented one month after birth, with AVRT refractory to triple AAD therapy and depressed left ventricular (LV) function on echocardiography.

*Patient 5* (female) arrived in cardiogenic shock at six weeks of age, with severely depressed LV function caused by a left-sided focal atrial tachycardia (AT). She required mechanical ventilation and inotropic support. Although heart rate was reduced with continuous intravenous esmolol, cardiac function did not improve.

*Patient 6* (male) had mitral valve stenosis and multiple ventricular septal defects (VSD), undergoing pulmonary artery banding and mitral valve repair at four months of age. The postoperative course was complicated by persistent AT unresponsive to adenosine. While AADs initially controlled the tachycardia, they had to be discontinued due to sinus node dysfunction. ATs with two different P wave morphologies frequently recurred in the following months, leading to RFCA before corrective surgery (debanding and closure of multiple VSDs).

#### Ablation procedure

**Fig. 1:**
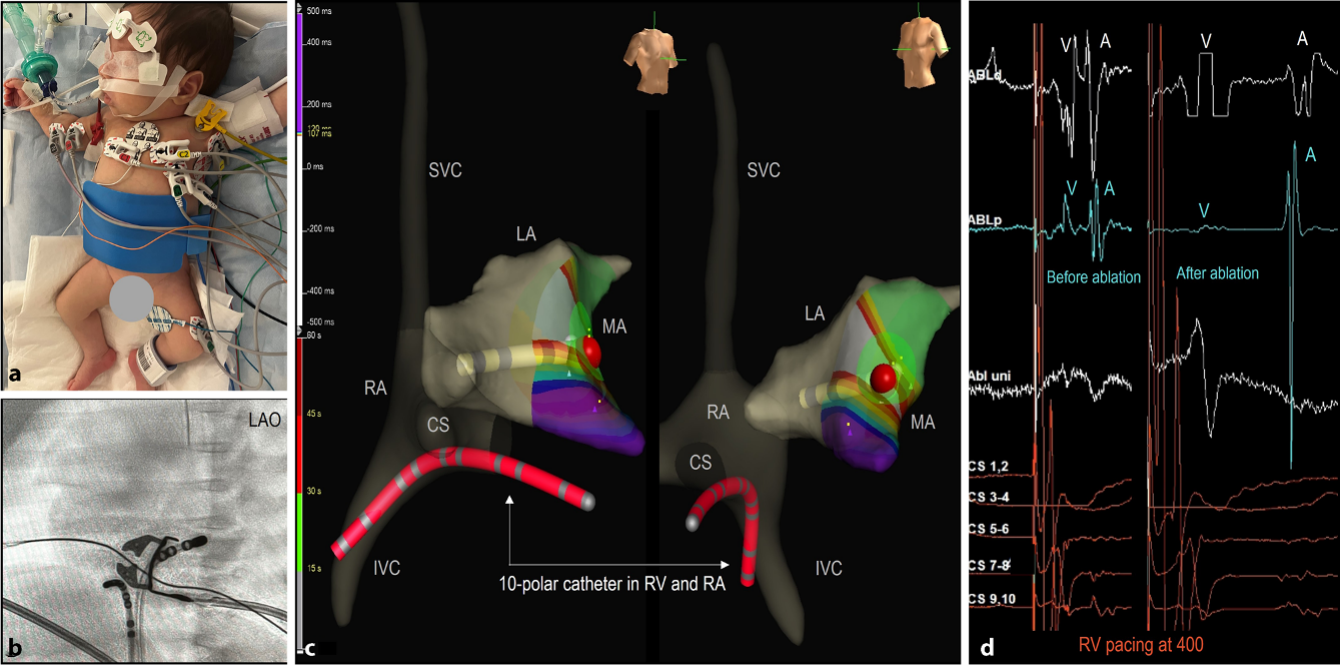
Adjustments to ablation procedures in infants. **a** Positioning of electrodes and patches: 3D electroanatomic system-, radiofrequency-, and ground-patches were cut to fit the infants’ torsos. **b** LAO fluoroscopic image of the 7 Fr RF catheter and the 5 Fr 10-pole steerable diagnostic catheter located in the RV and **c** 3D electroanatomical reconstruction of the right (*RA*) and left atria (*LA*) in RAO and LAO views showing the RF catheter and RF application (red dot) in the mitral annulus (*MA*) for a left sided concealed accessory pathway. A single 5 Fr 10-pole steerable catheter (red) was used for pacing and recording of the right atrium, His, and right ventricle (*RV*), reducing the number of required intracardiac catheters. **d** Intracardiac signals recorded during RV pacing before and after ablation, demonstrating a left-sided accessory pathway with the first atrial activation in the distal CS. Almost fused VA signals (ablation distal) were separated after ablation. *A* atrial, *CS* coronary sinus, *IVC* inferior vena cava, *LAO* left anterior oblique, *RAO* right anterior oblique, *SVC* superior vena cava, *V* ventricular

All procedures were conducted under general anaesthesia using an electroanatomic mapping (EAM) system (EnSite Precision, Abbott, St Paul, MN, USA). The surface electrodes and patches required modification to fit the infants’ torso (Fig. 1). Heparin was administered intravenously at a dose of 100 IU/kg. In most cases, a single 5 Fr ten-pole steerable catheter was used for pacing and recording of the right atrium (RA), His, and right ventricle (RV) signals (Fig. 1). Only in postoperative patient 6, two diagnostic catheters were placed in the coronary sinus and at the His position. When multiple catheters were needed, echo-guided vascular access was obtained through both femoral veins. AADs were discontinued 12 to 24 h before the procedure. Mapping was performed during SR in case of preexcitation, or during orthodromic AVRT and/or ventricular pacing. The procedural findings and techniques are summarized in Tab. 2, supplementary material.

In all cases, except for patient 6, mapping and ablation were started with a 5 Fr radiofrequency (RF) catheter (Therapy, M curve, 4 mm tip, Abbott, St Paul, MN, USA). However, due to poor steerability and/or low power related to high impedance during radiofrequency delivery, in three out of five cases, the catheters were exchanged for 7 Fr RF catheters (AlCath FullCircle, S curve, 4 mm gold-tip, Biotronik, Berlin, Germany—power setting 30 W, target temperature 50–55 °C) or 7 Fr irrigated tip RF catheters (AlCath Flux eXtra, S curve, 3.5 mm gold-tip, Biotronik, Berlin, Germany—power setting 20–25 W).

Patients 1 and 3 had an AVRT involving a concealed right lateral AP (Cycle length (CL) 370 ms) and an overt right posterior AP (CL 300 ms), respectively. Patients 2 and 4 both had AVRT with concealed left posterior and lateral APs (CL 270 ms, 280 ms) respectively, which were mapped through a patent foramen ovale (PFO) (Fig. 1). All four APs were successfully interrupted. However, in patient 1, accessing the right lateral AP via the inferior vena cava proved challenging, likely leading to suboptimal tissue contact and subsequent recurrence of AVRT within a few days. A second RFCA procedure from the left jugular vein resulted in improved contact and successful ablation. Similarly, patient 2 initially underwent RFCA with a 5 Fr RF catheter at relatively low power settings (20 Watts), but AVRT recurred within a week. A second RFCA using a 7 Fr RF catheter (30 Watts) provided better contact at the mitral valve annulus and was successful.

**Fig. 2:**
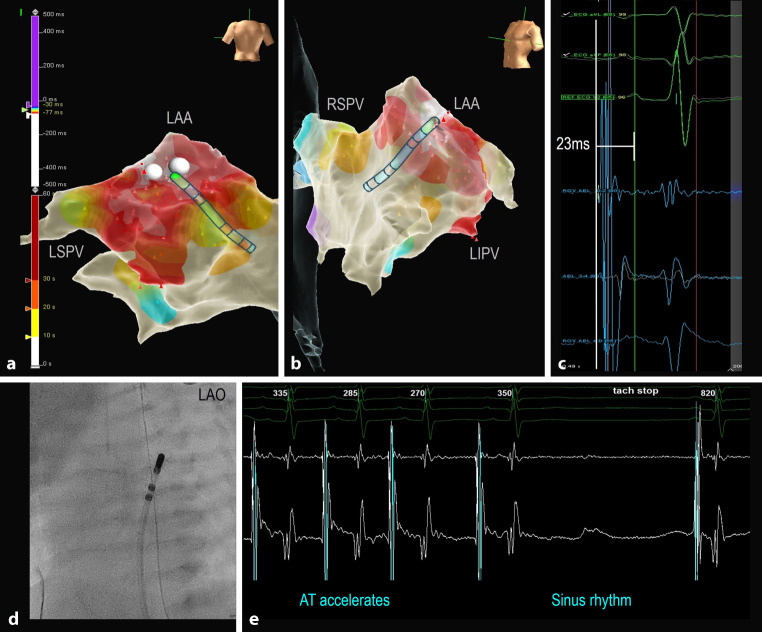
Focal atrial tachycardia (*AT*). Local activation map in PA (**a**) and LAO (**b**) views showing the location of the AT focus within the left atrial appendage (*LAA*). LAO fluoroscopic image (**d**) demonstrating the single 7 Fr intracardiac mapping/ablation catheter, which was advanced across the PFO to the left atrium and LAA. The onset of the earliest atrial activation recorded in the mapping/ablation catheter (**c**) precedes the peak of the reference P wave on the surface ECG by 23 ms. RF applications done in this location (**a,** **b**) resulted in acceleration and termination of the AT in less than 3 s (**e**). *RF* radiofrequency, *LAO* left anterior oblique, *LIPV/LSVP* left inferior/superior pulmonary vein, *PA* posteroanterior, *PFO* persistent *foramen ovale*, *RSPV* right superior pulmonary vein

Patient 5 presented with a left-sided incessant focal AT (CL 280–350 ms) and underwent mapping through the PFO using the P wave on the surface ECG as reference. The atrial focus was located in the small left atrial appendage and was successfully ablated with a 7 Fr irrigated tip catheter due to low power from impedance rise with both the non-irrigated 5 and 7 Fr catheters (see Fig. 2).

**Fig. 3:**
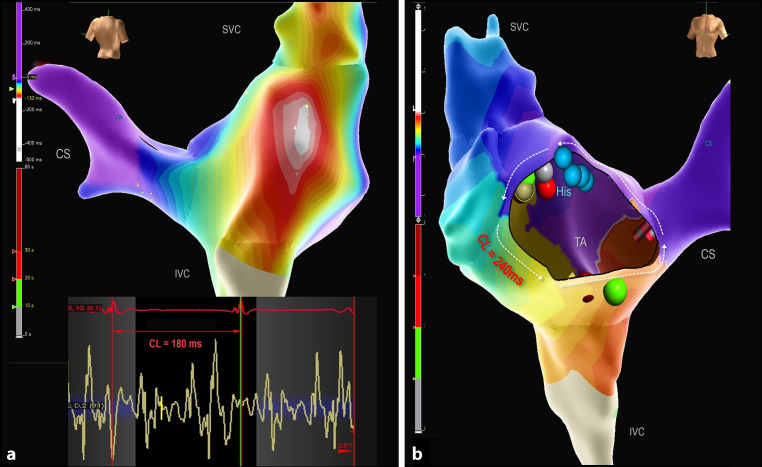
3D local activation maps of the right atrium showing two different SVT mechanisms in an infant with mitral valve stenosis and multiple ventricular septal defects. **a** Modified postero-anterior view shows a micro-reentrant tachycardia with a cycle length (*CL*) of 180 ms related to a very fragmented area in the upper part of the atriotomy scar as depicted by the intracardiac signals recorded by the mapping/ablation catheter. **b** Anteroposterior (right side) view showing a cavotricuspid isthmus (*CTI*) dependent counterclockwise atrial flutter with a CL of 240 ms. CTI was blocked with 3 RF applications (one green dot and one red dot partly visible at CTI position). Blue dots demarcate His position; the green, red, and grey dots next to the blue His dots, mark the location of the micro re-entrant tachycardia on the posterior wall. *CS* coronary sinus, *IVC* inferior vena cava, *SVC* superior vena cava, *TA* tricuspid annulus

Patient 6 exhibited two types of atrial tachycardias: a very fast focal micro-reentrant tachycardia (CL 180 ms) associated with a fragmented area in the upper part of the right atriotomy scar, and a cavotricuspid isthmus (CTI) dependent counterclockwise atrial flutter (CL 240 ms). The focal RA tachycardia was addressed first, and followed by CTI ablation, resulting in bidirectional CTI block (Fig. 3).

Overall, the mean procedural time (skin to skin) was 110 ± 5 min, the mean fluoroscopy time was 9 ± 7 min, and the mean dose area product was 275 mGycm^2^. The mean RF time was 170 (IQR 180–273) sec. Fluoroscopy was used less restrictively than in paediatric (≥ 5 years of age) ablation procedures for safety reasons to minimize the risk of perforations and other cardiac injuries in the small hearts.

### Follow-up

AAD therapy was discontinued after the procedure. No vascular complications occurred. Echocardiograms performed immediately after the procedures and during follow-up showed no pericardial effusion, visible thrombi, or valve damage. Patient 3, the smallest infant at 3 kg, had mild mitral valve regurgitation immediately after the second procedure using the 7 Fr catheter, which completely resolved after three weeks; all patients were extubated either after the procedure or the following day, and ECGs showed no signs of ischemia. Aspirin was administered at a dose of 1–2 mg/kg/day for three months. Cardiac function of patient 4 normalized within hours. Patient 5, who presented with severe TIC before RFCA, showed improved cardiac function after the procedure, with complete recovery noted three months later without the need for heart failure medication. No tachycardia recurrences were observed during a mean follow-up of 11 ± 6 months.

## RFCA in newborns and infants: review of the literature

AVRT is the most common type of SVT in neonates and infants, while focal AT and permanent junctional reciprocating tachycardia are less common chronic forms of SVT. Postoperative atrial flutter within the first year after neonatal CHD surgery, as seen in case 6, is extremely rare. Generally, these conditions have a favourable prognosis and often resolve spontaneously over time, though RFCA may be necessary in selected cases [2, 3, 5, 13, 14]. At our institution, we perform approximately 80 to 100 pediatric ablation procedures annually; however, RFCA has only been conducted in thirteen infants under one year of age in the past 20 years, including this recent consecutive series of six patients. The youngest was a premature newborn, weighing 1.9 kg, with an incessant AVRT (not included in this series) [15].

The number of RFCA in neonates and infants is relatively high at our institution, as we receive referrals from other centers in the Netherlands for the treatment of fetuses and neonates with drug-resistant SVT. These referrals are made for direct fetal therapy by intra-umbilical anti-arrhythmic drug treatment or postnatally for ablation, if necessary. However, based on the population-based study of Turner et al. [1] (incidence of infant SVT 22 cases per 100,000 live births), approximately 800 cases of neonatal SVT can be expected over 20 years in the Netherlands. The 13 infants who underwent ablation in our centre make up around 1 to 2% of the total infant SVT cases in the Netherlands.

The success of RFCA in infants is well-established in experienced medical centres and can be lifesaving [8, 16]. However, safety concerns remain, particularly for smaller infants and neonates, due to insufficient data and conflicting results. Initial findings from the Pediatric RFCA Registry suggested that the risk of severe complications—including complete AV block, perforation and/or pericardial effusion, valvular or coronary damage—is higher in children weighing less than 15 kg compared to older children [17]. This was confirmed in subsequent research [9]. Contributing factors include the smaller heart size, proximity of critical structures to the arrhythmia substrate, limited vascular access, thin-walled vessels and cardiac chambers, and catheter designs that are not ideal for this population. Severe complication rates as high as 4–8% have been reported in the youngest patients [13, 18–20], although these were not significantly different compared to older children, possibly due to the relatively small sample size in this age group. Nonetheless, there is general agreement that the risk of severe complications is usually higher in the youngest patients [8, 21].

While AV valve damage caused by RF energy or coronary lesions is a major but infrequent complication in infants [12] mild and transient AV valve regurgitation—particularly from the mitral valve—is relatively common following paediatric ablation procedures, as also evidenced in one of our cases, which completely resolved during follow-up [22–24].

Animal studies highlight the concerns regarding RF ablation in small hearts. Early research involving RF use in lambs showed significant expansion of lesions in the atria and ventricular free wall myocardium as the animals grew, while demonstrating only a limited increase in RF-equivalent lesions in the AV annuli [17, 25]. Similar findings were observed when cryo-energy and RF energy were applied to piglets, as AV groove lesions did not significantly increase with either energy modality. This offers some reassurance regarding the safety of AVRT ablation in infants [11]. However, the close proximity of the right and left circumflex coronary arteries to the endocardial AV annuli remains a concern [12, 22, 26]. Studies in piglets using RF ablation at the tricuspid and mitral annulus have shown a high incidence of both acute and late coronary lesions [27]. Notably, these lesions appeared to be independent of the number of RF applications and the maximum temperature achieved (60 °C) [28]. In contrast, cryoablation studies in animals with immature myocardium reported a significantly lower risk of coronary lesions [29–31].

Given these data, RF energy should be used with caution in infants. For example, using 5 Fr RF catheters and ablation cycles of 15–30 s with a low power limit of 20–30 W and temperature-controlled mode of 55 °C may reduce risks [15, 21, 25, 32, 33]. However, due to the limited steerability of the flexible 5 Fr design, we often had to switch from 5 Fr ablation catheters to 7 Fr catheters. Based on our experience, we now prefer to start with a 7 Fr small curve RF catheter. Prioritizing safety by using smaller RF catheters in combination with lower power settings (less than 50 W), along with shorter RF application durations, may reduce the risk of complications, but this approach can compromise efficacy, as some studies suggest a higher recurrence rate [34]. In one case, we also initially achieved successful ablation of a left posterior AP using a 5 Fr catheter with low RF energy (55 °C, max 20 Watts), but it recurred within a week. For the redo procedure, we used a 7 Fr RF catheter for better stability, with low temperature and power settings (30 Watts) to permanently interrupt the pathway. Cryoablation is potentially safer than RFCA and our first-line approach for ablating AV nodal re-entrant tachycardia and APs near the His region in children. However, while cryoablation appears particularly promising for infants, the cryo-catheter’s long radial curvature and limited flexibility make it challenging and precarious to navigate within small cardiac chambers. As a result, RFCA remains the preferred option in most cases [23, 35–37].

Another adjustment to ablation procedures in infants to avoid vascular damage and cardiac perforation is to reduce the number of catheters [7, 15, 19]. In young infants, an ablation catheter and an additional diagnostic catheter are typically sufficient, utilizing both the left and right femoral vein. A second diagnostic catheter can be positioned in the esophagus to replace the RA catheter or, as we did in most cases, a single 5 Fr six- or ten-pole catheter can be placed in the RV apex for pacing and recording RA, His bundle, and RV signals. In infants with Wolff-Parkinson-White syndrome or focal atrial tachycardia, a single RF catheter may suffice [15]. If the tachycardia mechanism is not easily identifiable, multiple catheters may be necessary, and additional vascular access, such as the jugular vein, should be considered. For left-sided substrates, an antegrade approach through a PFO or via transseptal puncture is recommended; we prefer using a J-shaped transseptal guidewire (SafeSept, Pressure Products, Inc., USA) or a RF wire to minimize the risk of complications during the puncture. In infants, the retrograde approach should be avoided due to the increased risk of arterial and aortic valve complications.

EAM systems significantly reduce radiation exposure by providing real-time visualization and guidance for catheters, as well as enabling anatomical reconstruction. The EnSite Precision system (Abbott, St. Paul, MN, USA), in particular, supports mapping and ablation catheters smaller than 7 Fr, making it potentially more suitable for newborns and infants. However, challenges remain in this age group, such as the large size of adhesive patches. It is important to carefully plan the placement of ECG electrodes, impedance field patches, and RF grounding pads to prevent suboptimal data acquisition due to patch overlap. Another concern is the heightened risk of signal and EAM distortion in very small patients, which may arise from limitations in field scaling or changes in impedance fields during procedures [38]. Most paediatric ablation programs, including ours, routinely utilize EAM systems and aim for (near) zero fluoroscopy. However, we believe that fluoroscopy remains necessary in these very small hearts to mitigate the risk of complications such as atrial or ventricular perforation, in addition to monitoring of endocardial signals.

Given the specific requirements for the use of catheters, energy settings, and EAM mapping system in neonates and infants, we think these procedures should only be performed by operators who have experience in interventional catheterisation in this young age group and electrophysiological procedures in older children. These procedures should therefore be embedded in a larger program with a dedicated team for interventional and electrophysiological procedures in children.

## Conclusion

RFCA in infants with SVT has advanced significantly due to improvements in clinical experience, periprocedural care, and technologies like EAM systems. However, uncertainty persists regarding the safety of this procedure in infants under 1 year of age. Given the potential risks associated with low body weight in newborns and infants, RFCA should be reserved for exceptional cases, such as life-threatening or drug-refractory arrhythmias that are unresponsive to amiodarone and combinations of AAD, and performed in highly experienced centers by qualified operators.

The decision to perform RFCA for SVTs in this age group requires careful consideration of several factors, including the technical challenges, the likelihood of success, potential for acute and long-term complications, the effectiveness of pharmacological treatment, the potential for spontaneous resolution, side effects of prolonged drug therapy, and prolongedintensive care stays.

## Supplementary Information

Table 2. Electrophysiologic findings, procedural techniques.
