# Supplementary material for: Radiofrequency ablation of supraventricular tachyarrhythmias in newborns and infants: why, when, and how?
Source: Neth Heart J. 2025 Sep 17;33(11):335–42. doi: 10.1007/s12471-025-01985-w (PMC12549470; doi:10.1007/s12471-025-01985-w)
Supplement: Supplementary file 1 — Table 2. Electrophysiologic findings, procedural techniques. [file 12471_2025_1985_MOESM1_ESM.docx]

**Table 2. Electrophysiologic findings, procedural techniques.**

| Patient | | | Tachycardia mechanism | | Cycle length  (ms) | | Number and characteristics of catheters | | RF energy exposure | | | Procedure duration  (min) | Fluoroscopy | |
| --- | --- | --- | --- | --- | --- | --- | --- | --- | --- | --- | --- | --- | --- | --- |
|  |  |  |  |  |  |  |  |  | T (ºC) | W | Time (sec) |  | Duration  (min) | PDA  (mGycm^2^) |
| 1 | 1^st^ RFCA | | Right lateral AP | | 370 | | 2 | -Abbott M curve (5Fr) >> Biotronik S curve, irrigated (7Fr)  -10-pole, Inquiry, Abbott (5Fr) | 35 | 15 | 400 | 240 | 23 | 530 |
|  | Re-do | |  |  | 420 | | 2 | - Biotronik S curve, irrigated (7Fr)  -10-pole, Inquiry, Abbott (5Fr) | 35 | 20 | 120 | 300 | 11 | 250 |
| 2 | 1^st^ RFCA | | Left posterior AP | | 270 | | 2 | -Abbott M curve (5Fr)  -10-pole, Inquiry, Abbott (5Fr) | 59 | 30 | 119 | 196 | 1 | 240 |
|  | Re-do | |  |  | No SVT induction | | 2 | -Biotronik S curve (7Fr)  -10-pole, Inquiry, Abbott (5Fr) | 57 | 30 | 79 | 180 | 1 | 205 |
| 3 | | | Right posterior AP | | 300 | | 2 | -Abbott M curve (5Fr) >> Biotronik S curve  -10-pole, Inquiry, Abbott (5Fr) | 47 | 40 | 152 | 240 | 6 | 221 |
| 4 | | | Left lateral AP | | 280 | | 2 | -Abbott M curve (5Fr)  -10-pole, Inquiry, Abbott (5Fr) | 54 | 30 | 60 | 192 | 3 | 168 |
| 5 | | | Focal AT from the LAA | | 280-350 | | 1 | -Abbott M curve (5Fr) >> Biotronik S curve, irrigated (7Fr) | 37 | 20 | 135 | 180 | 13 | 288 |
| 6 | | | CTI dependent AFL  Scar-related micro re-entrant tachycardia (RA lateral wall) | | 180-240 | | 3 | -Biotronik S curve  -10-pole, Inquiry, Abbott (5Fr)  -4-pole, Viking, Boston Scientific (5Fr) | 52 | 30 | 330 | 273 | 10 | 299 |
|  | |  | |  | |  |  |  |  |  |  |  |  |  |

AFL – atrial flutter; AP – accessory pathway; AT – atrial tachycardia; CTI – cavotricuspid isthmus; LAA – Left atrium appendage; min – minutes; PDA – product dose area; RA – Right atrium; RFCA – radiofrequency catheter ablation; RF – radiofrequency; sec – seconds; T – temperature (ºC: degrees Celsius); W – watt.
